# Analysis of polio vaccination status in a cohort of live births in 2017 and 2018 in Brazilian cities: a national vaccination coverage survey

**DOI:** 10.1590/S2237-96222024v33e20231303.en

**Published:** 2025-01-10

**Authors:** Alessandra Lucchesi de Menezes Xavier Franco, Ana Paula França, José Cássio de Moraes, Manoel Carlos Sampaio de Almeida Ribeiro, ICV 2020 Group, Adriana Ilha da Silva, Alberto Novaes Ramos, Ana Paula França, Andrea de Nazaré Marvão Oliveira, Antonio Fernando Boing, Carla Magda Allan Santos Domingues, Consuelo Silva de Oliveira, Ethel Leonor Noia Maciel, Ione Aquemi Guibu, Isabelle Ribeiro Barbosa Mirabal, Jaqueline Caracas Barbosa, Jaqueline Costa Lima, José Cássio de Moraes, Karin Regina Luhm, Karlla Antonieta Amorim Caetano, Luisa Helena de Oliveira Lima, Maria Bernadete de Cerqueira Antunes, Maria da Gloria Teixeira, Maria Denise de Castro Teixeira, Maria Fernanda de Sousa Oliveira Borges, Rejane Christine de Sousa Queiroz, Ricardo Queiroz Gurgel, Rita Barradas Barata, Roberta Nogueira Calandrini de Azevedo, Sandra Maria do Valle Leone de Oliveira, Sheila Araújo Teles, Silvana Granado Nogueira da Gama, Sotero Serrate Mengue, Taynãna César Simões, Valdir Nascimento, Wildo Navegantes de Araújo

**Affiliations:** 1 Centro de Vigilância Epidemiológica “Prof. Alexandre Vranjac”, Coordenadoria de Controle de Doenças, São Paulo, SP, Brazil; 2 Faculdade de Ciências Médicas da Santa Casa de São Paulo, Departamento de Saúde Coletiva, São Paulo, SP, Brazil; 3 Coordenadoria de Controle de Doenças, Secretaria de Estado da Saúde de São Paulo, São Paulo, SP, Brazil; Universidade Federal do Espírito Santo, Vitória, ES, Brazil; Universidade Federal do Ceará, Departamento de Saúde Comunitária, Fortaleza, CE, Brazil; Faculdade Ciências Médicas Santa Casa de São Paulo, São Paulo, SP, Brazil; Secretaria de Estado da Saúde do Amapá, Macapá, AP, Brazil; Universidade Federal de Santa Catarina, SC, Brazil; Organização Pan-Americana da Saúde, Brasília, DF, Brazil; Instituto Evandro Chagas, Belém, PA, Brazil; Faculdade de Ciências Médicas Santa Casa de São Paulo, Departamento de Saúde Coletiva, São Paulo, SP, Brazil; Universidade Federal de Mato Grosso, Cuiabá, MT, Brazil; Universidade Federal do Paraná, Curitiba, PR, Brazil; Universidade Federal de Goiás, Goiânia, GO, Brazil; Universidade Federal do Piauí, Teresina, PI, Brazil; Universidade de Pernambuco, Faculdade de Ciências Médicas, Recife, PE, Brazil; Instituto de Saúde Coletiva, Universidade Federal da Bahia, Salvador, BA, Brazil; Secretaria de Estado da Saúde de Alagoas, Maceió, AL, Brazil; Universidade Federal do Acre, Rio Branco, AC, Brazil; Universidade Federal do Maranhão, Departamento de Saúde Pública, São Luís, MA, Brazil; Universidade Federal de Sergipe, Aracaju, SE, Brazil; Secretaria Municipal de Saúde, Boa Vista, RR, Brazil; Fundação Oswaldo Cruz, Mato Grosso do Sul, Campo Grande, MS, Brazil; Fundação Oswaldo Cruz, Escola Nacional de Saúde Pública Sergio Arouca, Rio de Janeiro, RJ, Brazil; Universidade Federal do Rio Grande do Sul, Porto Alegre, RS, Brazil; Fundação Oswaldo Cruz, Instituto de Pesquisa René Rachou, Belo Horizonte, MG, Brazil; Secretaria de Desenvolvimento Ambiental de Rondônia, Porto Velho, RO, Brazil; Universidade de Brasília, Brasília, DF, Brazil

**Keywords:** Cobertura de Vacunación, Vacunas contra Poliovirus, Poliomielitis, Salud Infantil, Encuestas Epidemiológicas, Vaccination Coverage, Poliovirus Vaccines, Poliomyelitis, Child Health, Epidemiological Surveys

## Abstract

**Objective:**

To describe the polio vaccination status in 26 state capitals, the Federal District, and 12 municipalities in Brazil, among children born between 2017 and 2018.

**Methods:**

This was a population-based household survey conducted from 2020 to 2022, which assessed polio vaccination coverage in children, considering valid, administered, and timely doses by municipality.

**Results:**

Data were collected from 37,801 children. Vaccination coverage for the complete valid dose schedule was 87.5% (95%CI 86.2;88.7), dropping to 79.6% (95%CI 78.1;81.0), when the booster dose was considered. The dropout rate was 4.5% for the complete schedule, and 11.7% for the first booster. There was no correlation between campaign implementation and high coverage.

**Conclusion:**

Vaccination coverage for the complete valid dose schedule and the first booster did not meet the 95.0% target. Regional disparities and the association between vaccination coverage and social indicators should be taken into consideration in strategies to increase coverage.

## Study contributions

**Main results:**

Vaccination coverage for the complete poliomyelitis schedule was 87.5% (95%CI 86.2;88.7), with a reduction to 79.6% (95%CI 78.1;81.0) for the first booster dose. Dropout rate was 4.5% for the complete schedule, and 11.7% for the first booster dose.

**Implications for services:**

Checking vaccination booklets helps identify and minimize factors associated with non-vaccination, which should be considered when defining effective strategies aimed at increasing vaccination coverage.

**Perspectives:**

Understanding vaccination coverage guides the development of actions that support the improvement of vaccination coverage, consequently reducing the number of susceptible individuals and helping to control vaccine-preventable diseases.

## RESUMEN

**Objetivo:**

Describir el estado vacunal contra la poliomielitis en 26 capitales estatales, Districto Federal y 12 municipios de Brasil, en niños nacidos en 2017-2018.

**Métodos:**

Encuesta de hogares de 2020 a 2022, en la cual se evaluó la cobertura vacunal contra la poliomielitis, considerando doses válidas, aplicadas y oportunas por municipios.

**Resultados:**

Se recogieron datos de 37.801 niños. La cobertura vacunal del esquema completo de dosis válidas fue 87,5% (IC95% 86,2;88,7), cayendo a 79,6% (IC95% 78,1;81,0), considerando el refuerzo de dosis. La tasa de abandono fue del 4,5% para la pauta completa y del 11,7% para el primer refuerzo. No hubo correlación entre realizar una campaña y una alta cobertura.

**Conclusión:**

La cobertura de vacunación en esquema completo y en primer refuerzo no alcanzó la meta del 95,0%. Las diferencias regionales y la asociación de la cobertura vacunal con indicadores sociales deben considerarse en las estrategias para aumentarla.

## INTRODUCTION

Since 2016, vaccination coverage for vaccines included in the childhood vaccination schedule has been decreasing, reflecting the unsatisfactory result of this indicator for the polio vaccine in Brazil. In 2020, 2021 and 2022, national vaccination coverage for the complete schedule with first booster was 73.0%, 65.8% and 72.5%, respectively, falling short of the recommended target of 95.0%.^1^ These results may be associated with various factors, such as operational challenges, worsening socioeconomic conditions and vaccine hesitancy.^2^ The introduction of the polio vaccine in Brazil dates back to 1961.^3^ In 2016, the use of the inactivated polio vaccine (IPV) was incorporated into the vaccination schedule, with three doses administered during the child’s first year of life (at 2, 4 and 6 months old), followed by two booster doses of the bivalent oral polio vaccine (bOPV),^4^ at 15 months and 4 years of age, as well as its administration during annual vaccination campaigns.^5^ However, the aforementioned vaccination schedule will undergo changes in 2024, as the World Health Organization (WHO) recommends the use of IPV whenever possible ,^6^ and, therefore, booster doses will be administered with IPV rather than bOPV.^5,7^

It is worth highlighting that poliomyelitis is an acute viral infectious disease primarily affecting children under 5 years of age, and in 1% of cases, the disease causes sudden onset of asymmetric flaccid paralysis in the lower limbs, with preserved sensation, and areflexia in the affected region.^8^ Transmission occurs most frequently through direct contact with feces or secretions from the mouth of sick people or carriers.^8,9^ Motor deficits often progresses within three days, with 5% to 10% of cases resulting in death due to respiratory muscle paralysis, and ^10^ and one in every 200 infections leads to irreversible paralysis.^8^

It is worth noting that in 1988, the 41^st^ World Health Assembly adopted a resolution for the global eradication of poliomyelitis, which led to the creation of the Global Polio Eradication Initiative (GPEI).^11^ This initiative enhanced the capacity to combat other infectious diseases and to improved surveillance and immunization efforts,^12^ which contributed to the reduction of cases, given that currently 80% of the world’s population lives in regions certified as polio-free.^13^

Brazil was certified as polio-free in 1994; however, there is a risk of imported cases,^14^ as there are countries with endemic transmission, such as Afghanistan and Pakistan.^15,16^ Nevertheless, over 25 countries have detected cases of wild poliovirus type 1 or vaccine-derived poliovirus, posing a potential risk of international spread.^16^ It is noteworthy that, as long as there is an infected child, susceptible children in all countries are at risk of contracting poliomyelitis,^15^ highlighting the need to maintain high vaccination coverage,^9,11^ a fact that underscores the importance of conducting studies that help understand the vaccination situation and guide actions to achieve better results. 

The objective of this study was to describe the polio vaccination status in 26 state capitals, the Federal District and 12 municipalities in Brazil, in children born in 2017 and 2018.

## METHODS

This was a population-based household survey, conducted from a cohort of live-born children in 2017 and 2018, residing in the urban area of the 26 state capitals, the Federal District and 12 municipalities with over 100,000 inhabitants, namely: Campinas/São Paulo state, Caruaru/Pernambuco state, Imperatriz/Maranhão state, Joinville/Santa Catarina state, Londrina/Paraná state, Petrópolis/Rio de Janeiro state, Rio Grande/Rio Grande do Sul state, Rio Verde/Goiás state, Rondonópolis/Mato Grosso state, Sete Lagoas/Minas Gerais state, Sobral/Bahia state and Vitória da Conquista/Bahia state.^2^

### Data collection

Data collection was carried out between September 2020 and March 2022, taking into account the social distancing periods implemented in each location. Data on the dates of administration of each vaccine during the first 24 months of life, were obtained considering the vaccines administered routinely, in both public and private services, a well as those administered during campaigns.

The vaccination booklets were photographed, read and transcribed into the research database by nurses with experience in the activities of the National Immunization Program (*Programa Nacional de Imunizações* - PNI). Those booklets not found in the household were retrieved from the PNI Information System (*Sistema de Informação do PNI* - SI-PNI). In addition, a structured questionnaire was administered, containing closed-ended questions related to the sociodemographic data of the child; the reproductive and sociodemographic data of the mother; household and family consumption information; and the child’s vaccination data. More details on the data collection instrument and field strategies are described in the methodological reference article.^2^

### Sampling

The sample design considered separate surveys for each city. Thus, the following were considered for the calculation: a hypothetical population of 1 million live births; estimated prevalence of vaccination coverage = 70%, margin of error = 5%; z = 1.96 for a 95% confidence interval (95% CI); and a design effect of 1.4, resulting in a total of 452 children per survey.

Depending on the population size of live births, between one and four surveys were conducted per municipality, with only one survey being conducted in 15 cities (four capitals and 11 inland cities), two surveys in nine capitals, three surveys in four capitals and four surveys in nine capitals, he Federal District and one inland city.

The sampling procedure begins with the definition of socioeconomic strata created through cluster analysis, based on socioeconomic indicators (average income of household heads, proportion of literate heads of household and proportion of heads of household with income greater than or equal to 20 minimum wages) from the urban census tracts of each city, according to data from the 2010 Demographic Census.

The cluster analysis generated four strata (A to D) of census tracts with distinct socioeconomic characteristics. Stratum A is related to high-income socioeconomic groups; B, upper-middle income; C, lower-middle income; and D, low-income.² Children from the cohorts of interest living in each tract were estimated through georeferencing of addresses contained in the Live Birth Information System (*Sistema de Informação sobre Nascidos Vivos* - SINASC) and projections based on the distribution observed in the 2010 Census.

The sectors were grouped by proximity (clusters) and expected number of children, so that each cluster contained three times the number of children to be included in the sample. The clusters were systematically selected to cover the entire geographic area. Using maps of the clusters and the list of addresses obtained from SINASC, the interviewers covered the area identifying children from the cohorts until the pre-established number was reached for each stratum in each city, as described in the methodological article of the study.^2^

### Indicators and data analysis

To calculate vaccination coverage, the number of live births registered in SINASC in 2017 and 2018 was used as the denominator; for numerators, different criteria were applied to consider doses administered (dose administered regardless of timing) , valid doses (dose administered considering the timing, in relation to the child’s date of birth, and observing the interval between doses) and timely (doses recommended according to the vaccination schedule, taking into account the minimum and maximum ages for each vaccine and adequate intervals between doses),^17^ detailed below:

- Vaccination coverage at 12 months
  - Doses administered
    - Dose 1: combination of the first dose of IPV + hexavalent vaccine (acellular pertussis, IPV, hepatitis B and *Haemophilus influenzae* type B) + acellular (acellular pertussis and IPV);
    - Dose 2: combination of the second dose of IPV + hexavalent vaccine + acellular vaccine;
    - Dose 3: combination of the third dose of IPV + hexavalent vaccine + acellular vaccine.
  - Valid doses
    - Dose 1: first dose administered at 42 days or older
    - Dose 2: second dose administered at least 30 days after the first dose;
    - Dose 3: third dose administered at least 30 days after the second dose.
  - Timely doses
    - Dose 1: first dose administered between 42 and 89 days;
    - Dose 2: second dose administered between 70 and 151 days;
    - Dose 3: third dose administered between 98 and 212 days.
- Vaccination coverage considering the first booster dose
  - Doses administered
    - Dose 1: combination of the first dose of OPV + fourth dose of IPV administered ≥ 12 months.
  - Valid doses of OPV
    - Dose 1: first dose administered at 365 days or more after having received the previous three IPV doses.
  - Timely doses of OPV
    - Dose 1: first dose administered between 365 and 486 days, having received the previous three IPV doses.

The following were also considered:

- Dropout rate indicator for complete IPV vaccination schedule: difference between dose 1 IPV administered and dose 3 IPV administered/dose 1 IPV administered*100.
- Dropout rate indicator for booster dose: difference between dose 1 IPV administered and dose 1 OPV (first booster)/ dose 1 IPV administered*100.

Vaccination coverage indicators were analyzed in general, considering the different dose criteria. Valid dose indicators were used to analyze coverage behavior among capitals and municipalities, which was also used in the preliminary investigation of the association between polio vaccination coverage and socioeconomic and demographic factors.

In order to analyze vaccination coverage, five groups were also established: satisfactory (equal to or greater than 95%); reasonable (90% to 94.9%); unsatisfactory (80% to 89.9%); critical (70% to 79.9%); and very critical (equal to or less than 69.9%). These groups were established based on the understanding that satisfactory coverage corresponds to the recommended target of 95%,^18^ and the other groups below this, so that, as the values move away from the recommended target, the greater the efforts of the municipality to reach the target, and the larger the susceptible population; consequently, the greater the risk of disease and transmission.

As the sample was stratified and clustered by census sector with disproportionate allocation, it was necessary to calculate and apply sample weights to each household interviewed for estimates. The analyses were performed by applying the definitions of weights, strata and clusters to calculate the estimates of vaccination coverage and their respective 95% confidence intervals, as well as correlations between coverage, first booster schedule and vaccination campaigns were analyzed by means of Spearman’s correlation test, using SPSS version 22.

### Ethical aspects

The research was approved by the Research Ethics Committees of the Instituto de Saúde Coletiva da Universidade Federal da Bahia, under opinion No. 3,366,818, on June 4, 2019, with Certificate of Submission for Ethical Appraisal (CAAE) 4306919.5.0000.5030; and of the Irmandade da Santa Casa de São Paulo, under opinion No. 4,380,019, on November 4, 2020, with CAAE 39412020.0.0000.5479. 

## RESULTS

The final sample consisted of 37,801 live births, after a loss corresponding to 6% of the total sample in each municipality. Thus, it was observed that vaccination coverage based on doses administered for IPV (complete schedule) was 88.0% (95%CI 86.7;89.1) and for OPV it was 81.2% (95%CI 79.8;86.7) (first booster dose) both of which are below the 95.0% expected by the Ministry of Health (Table 1).

**Table 1:** Polio vaccination coverage in (%) and 95% confidence intervals (95%CI), indicators for the complete schedule and the first booster dose, in the cohort of live births in 2017 and 2018, across the state capitals, in the Federal District and in 12 other municipalities, Brazil, 2020-2021 (n=37,801)

Indicator	Frequency	% (95%CI)
**Doses administered**		
1^st^ dose of inactivated polio vaccine	34,826	92.1 (91.1;93.0)
3^rd^ dose of inactivated polio vaccine	33,273	88.0 (86.7;89.1)
1^st^ dose of oral polio vaccine	30,746	81.2 (79.8;86.7)
**Valid doses**		
1^st^ dose of inactivated polio vaccine	34,688	91.5 (90.5;92.5)
3^rd^ dose of inactivated polio vaccine	33,064	87.5 (86.2;88.7)
1^st^ dose of oral polio vaccine	30,227	79.6 (78.1;81.0)
**Timely doses**		
1^st^ dose of inactivated polio vaccine	31,000	82.7 (81.4;84.0)
3^rd^ dose of inactivated polio vaccine	20.333	58.3 (56.5;60.1)
1^st^ dose of oral polio vaccine	15,045	41.6 (39.9;43.2)

This result indicates a 6.8 percentage point difference between the completion of the vaccination schedule and the first booster dose, recommended at 15 months old. It is noteworthy that the dropout rate for the complete IPV schedule based on doses administered was 4.5%, lower than the dropout rate related to the vaccination schedule with the first booster dose (11.7 %) (Table 1).

When criteria for considering valid doses were established, there was a reduction in coverage for the complete IPV schedule (87.5%; 95%CI 86.2;88.7) and OPV (79.6%; 95%CI 78.1;81.0). This result highlights a greater reduction in valid and timely dose coverage for OPV doses compared to the coverage of these doses for the schedule with IPV doses. For 1.7% of the children who received OPV, the doses administered were not considered valid, while for 0.6%, the third doses of IPV administered were not considered valid (Table 1).

When vaccination coverage was analyzed based on timeliness, there was a greater reduction than when coverage was based on administered and valid doses, both for IPV (58.3%; 95%CI 56.5;60.1) and for OPV (41.6%; 95%CI 39.9;43.2). It is found that, among the doses administered, 38.9% of the third doses of IPV were not administered between 98 and 212 days after the second dose of IPV, and that 51.1% of the first booster doses with OPV were administered between 365 and 486 days after completion of the IPV schedule (Table 1).

Regarding vaccination coverage of the first booster, the target was not reached in any of the cities analyzed, with the highest coverage being 93.3% (95%CI 90.7;95.2) in Joinville/Santa Catarina state, and the lowest being 61% (95%CI 57.3;67.9) in Florianópolis/Santa Catarina state (Table 2). From this perspective, considering the established groups, a concentration of 10.3% of the municipalities was observed in the *reasonable group*, with vaccination coverage ranging from 90.5% to 93.3%. In the *unsatisfactory group,* 53.8% of the municipalities were concentrated with coverage ranging from 80% to 89.1%; in the *critical group*, coverage ranged from 70.9% to 79.8%, accounting for 30.8% of the municipalities. Finally, in the *very critical group* , 5.1% of municipalities with coverage ranging from 61.0% (95%CI 53.7;67.9) to 62.2% (95%CI 54.3;69.5), were observed (Table 2).

**Table 2:** Vaccination coverage indicator in (%) and 95% confidence intervals (95%CI), for the inactivated polio vaccine (IPV) and the oral polio vaccine (OPV) administered routinely and in campaigns in the cohort of live births in 2017 and 2018, according to valid doses by capital, inland cities and the Federal District, Brazil, 2020-2021 (n = 37,801)

City	FU	Region	n	Coverage (3^rd^ IPV dose + 1^st^ OPV dose)		Campaign	
				Frequency	% (95%CI)	Frequency	% (95%CI)
Joinville	SC	S	460	409	93.3 (90.7;95.2)	258	62.2 (53.9;69.8)
Caruaru	PE	NE	462	422	91.8 (86.6;95.1)	302	61.2 (52.8;68-9)
Porto Velho	RO	N	451	398	91.2 (87.5;93.9)	161	39.6 (30.7;49.3)
Teresina	PI	NE	899	771	90.5 (86.1;93.6)	540	60.9 (53.8;67.6)
Imperatriz	MA	NE	465	423	89.1 (84.2;92.7)	190	36.6 (31.6;41.9)
Sete Lagoas	MG	SE	451	411	89.0 (82.8;93.2)	290	63.2 (52.7;72.5)
Londrina	PR	S	455	370	88.9 (81.7;93.5)	227	47.1 (41.5;52.8)
Salvador	BA	NE	1,818	1.550	88.4 (83.9;91.8)	721	43.0 (38.7;47.4)
Boa Vista	RR	N	395	334	88.1 (81.8;92.4)	172	46.9 (41.9;52.0)
Rio Verde	GO	CO	444	391	87.4 (77.2;93.4)	229	51.7 (42.2;61.1)
Cuiaba	MT	CO	814	703	87.3 (81.4;91.6)	411	52.2 (48.0;56.4)
São Luis	MA	NE	854	708	86.8 (81.2;90.9)	362	39.2 (28.8;50.7)
Sobral	CE	NE	465	356	85.9 (71.5;93.7)	225	67.2 (47.3;82.3)
Manaus	AM	N	1,826	1.546	85.8 (82.1;88.9)	988	55.7 (50.5;60.8)
Porto Alegre	RS	S	1,383	1.012	84.2 (78.8;88.4)	465	41.1 (35.4;47.0)
Rio Grande	RS	S	452	352	84.0 (72.2;91.4)	203	46.7 (31.0;63.1)
Palmas	TO	N	453	354	83.8 (77.3;88.7)	243	54.4 (47.4;61.2)
Petropolis	RJ	SE	468	415	82.8 (70.5;90.7)	236	44.4 (30.4;59.3)
Aracaju	IF	NE	900	733	82.7 (75.3;88.2)	552	62.4 (56.6;67.9)
Rio Branco	AC	N	451	361	82.7 (78.2;86.5)	160	34.1 (25.9;43.5)
Vitória da Conquista	BA	NE	455	336	82.1 (67.1;91.2)	193	50.0 (37.4;62.6)
Rondonopolis	MT	CO	449	353	81.3 (72.2;87.9)	196	46.2 (39.2;53.5)
Goiania	GO	CO	1.811	1.453	81.2 (73.8;86.9)	871	50.1 (42.8;573)
Brasilia	DF	CO	1.809	1.362	80.9 (77.3;84.1)	980	57.8 (53.5;62.1)
Maceio	AL	NE	929	744	80.0 (67.2;88.7)	466	46.7 (39.1;54.4)
Fortaleza	CE	NE	1.612	1.328	79.8 (74.3;84.3)	830	51.9 (47.0;56.7)
Belo Horizonte	MG	SE	1,863	1.404	78.9 (74.0;83.1)	1.011	59.5 (54.6;64.2)
Recife	PE	NE	1,689	1.381	78.9 (69.0;86.2)	940	53.6 (45.2;61.7)
São Paulo	SP	SE	1,539	1.250	77.8 (72.8;82.1)	669	44.0 (38.7;49.4)
Campo Grande	MS	CO	1.281	994	77.3 (71.2;82.4)	593	48.6 (42.7;54.5)
João Pessoa	PB	NE	904	705	75.8 (68.8;81.6)	492	53.0 (47.4;58.4)
Rio de Janeiro	RJ	SE	1,820	1.351	74.9 (70.3;78.9)	767	44.8 (39.0;50.8)
Campinas	SP	SE	1,774	1.481	74.5 (62.4;83.8)	826	42.8 (35.0;51.0)
Belém	PA	N	1.218	1.008	73.7 (63.7;81.7)	539	35.5 (27.7;44.1)
Natal	RN	NE	685	530	71.8 (60.5;80.9)	348	47.7 (39.7;55.9)
Curitiba	PR	S	1.192	880	71.5 (62.7;78.9)	538	50.9 (43.6;58.1)
Macapa	AP	N	878	624	70.9 (66.4;75.0)	238	26.2 (20.7;32.6)
Victória	ES	SE	788	504	62.2 (54.3;69.5)	458	44.6 (29.9;60.2)
Florianopolis	SC	S	739	520	61.0 (53.7;67.9)	291	32.6 (26.3;39.6)

Vaccination coverage for booster doses in the 12 large urban centers (São Paulo/São Paulo state, Brasília/Federal District, Rio de Janeiro/Rio de Janeiro state, Manaus/Amazonas state, Belém/Pará state, Fortaleza/Ceará state, Recife/Pernambuco state, Salvador/Bahia state, Belo Horizonte/Minas Gerais state, Curitiba/Paraná state, Goiânia/Goiás state and Porto Alegre/Rio Grande do Sul state) ranged from 71.5% (95%CI 62.7;78.9) to 88.4% (95%CI 83.9;91.8), with a higher concentration of these centers in the critical group (Table 2).

It was not possible to observe high coverage of campaign doses (Table 2), and the data show that, after performing the statistical test, there was no correlation between conducting campaigns and high vaccination coverage.

When socioeconomic strata were observed, none achieved the recommended vaccination coverage; stratum C showed the best coverage for valid IPV doses (89.1%; 95%CI 87.4;90.7); however, stratum D presented better coverage for the vaccination schedule with the first booster (84.8%; 95%CI 83.0;86.4), higher than stratum A [complete schedule – 79.1% (95%CI 74.2;83.2) and with first booster – 64.7% (95%CI 60.0;69.2)]. Furthermore, it is inferred that booster dose coverage varied more between strata [64.7% (95%CI 60.0;69.2) to 84.8% (95%CI 83.0;86.4)] than IPV coverage [79.1% (95%CI 74.2;83.2) to 89.1% (95%CI 87.4;90.7)] (Table 3).

**Table 3:** Vaccination coverage indicators in (%) and 95% confidence intervals (95%CI), for the inactivated polio vaccine (IPV) and the oral polio vaccine (OPV) in the cohort of live births in 2017 and 2018, across the capitals, inland cities and the Federal District, according to socioeconomic strata, Brazil, 2020-2021 (n = 37,801)

Stratum	n	Valid 3^rd^ IPV doses		Valid 3^rd^ IPV doses + Valid OPV doses	
		Frequency	% ( 95%CI)	Frequency	% ( 95%CI)
A	8.333	7,067	79,1 (74,2;83,2)	6.062	64,7 (60,0;69,2)
B	9,418	8.165	86,5 (82,9;89,4)	7.336	68,8 (63,9;73,3)
C	9,992	8.910	89,1 (87,4;90,7)	8,347	80,9 (78,4;83,1)
D	10,058	8,922	88,9 (87,1;90,5)	8,482	84,8 (83,0;86,4)

In this context, when analyzing the characteristics of the family, mother and child, it can be inferred that, for the complete IPV schedule, when considering the confidence intervals, vaccination coverage for strata according to consumer goods C (89.1%; 95%CI 87.3;90.7) and D (88.2%; 95%CI 86.4;89.7) is higher than for stratum B (87.3%; 95%CI 84.6;89.6), which, in turn, is higher than stratum A (81.8%; 95%CI 75.7;86.7). It is noted that vaccination coverage for those who receive the Bolsa Família Program benefit (90.9%; 95%CI 89.3;92.3) is higher than for those who do not receive it (86.3%; 95%CI 84.8;87.7) (Table 4).

**Table 4:** Vaccination coverage indicators in (%) and 95% confidence intervals (95%CI), for the inactivated polio vaccine (IPV) and the oral polio vaccine (OPV) in the cohort of live births in 2017 and 2018, across the capitals, cities and the Federal District, according to family, maternal and child characteristics, Brazil, 2020-2021 (n = 37,801)

Family, maternal and child characteristics	Valid 3^rd^ IPV doses		Valid 1^st^ OPV doses	
	Frequency	% (95%CI)	Frequency	% (95%CI)
**Mother’s race/skin color**				
White	13.195	87.0 (84.8;88.9)	11,471	75,1 (72,6;77,5)
Black	3.763	89.4 (86.7;91.6)	3.611	87,9 (85,3;90,1)
Mixed-race	14,909	88.3 (86.5;89.9)	14,054	83,1 (81,4;84,7)
Asian	324	89.4 (78.1;95.2)	275	70,0 (53,8;82,4)
Indigenous	111	90.0 (78.2;95.8)	101	85,3 (74,2;92,1)
Unknown	762	72.6 (63.4;80.2)	715	68,6 (59,8;76,3)
**Consumer goods stratum**				
A	1,622	81.8 (75.7;86.7)	1.066	43,3 (36,2;50,8)
B	7,822	87.3 (84.6;89.6)	6.621	69,8 (66,3;73,0)
C	10.318	89.1 (87.3;90.7)	9,923	85,7 (83,6;87,5)
D	12.321	88.2 (86.4;89.7)	11,714	85,0 (83,3;86,7)
Unknown	981	72.7 (64.5;79.6)	903	68,1 (59,5;75,6)
**Bolsa Família Program**				
Yes	9,089	90.9 (89.3;92.3)	8,662	86,8 (85,2;88,2)
No	23,866	86.3 (84.8;87.7)	21,457	77,1 (75,3;78,8)
Unknown	109	87.2 (75.7;93.8)	108	86,3 (76,0;92,6)
**Child’s sex**				
Male	16,993	87.5 (86.0;88.9)	15,540	80,5 (78,7;82,1)
Female	16,071	87.5 (85.8;89.0)	14,687	78,7 (76,6;80,7)
**Birth order**				
First-born	16.013	88.2 (86.5;89.6)	14,584	79,3 (77,2;81,3)
Second-born	10.613	86.4 (84.3;88.3)	9,667	80,0 (77,7;82,2)
Third-born	4.084	88.3 (85.8;90.5)	3,796	79,6 (76,1;82,7)
Fourth-born or later	2.331	87.0 (82.8;90.2)	2.156	79,6 (74,3;84,1)
**Mother’s age (years)**				
< 20	765	87.4 (80.4;92.1)	729	85,5 (79,6;89,9)
20-34	18,973	86.9 (85.3;88.3)	17,921	83,0 (81,4;84,4)
≥ 35	13.182	88.4 (86.6;90.0)	11,442	74,9 (72,2;77,5)
**Partner**				
Yes	24,820	87.8 (86.4;89.1)	22,450	78,6 (76,8;80,2)
No	7,327	87.9 (85.8;89.7)	6.909	84,0 (81,7;86,0)
Unknown	917	76.4 (68.3;82.9)	868	73,2 (65,2;79,9)
**Grandmother lives with the family**				
Yes	8,704	86.2 (84.0;88.1)	8.251	81,4 (79,0;83,7)
No	24.314	88.0 (86.6;89.3)	21,936	78,9 (77,1;80,6)
**Mother has a job**				
Yes	13,506	90.2 (88.7;91.4)	13,666	84,5 (82,8;86,1)
No	16,539	86.0 (84.0;87.7)	15,863	76,0 (73,9;78,0)
Unknown	735	73.5 (64.3;81.0)	698	69,7 (60,9;77,3)
**Mother’s education level (years)**				
≤ 8	2,871	87.5 (84.5;90.0)	2.736	82,3 (78,6;85,4)
9-12	4,828	87.0 (84.1;89.5)	4,627	84,6 (81,7;87,1)
13-15	13,608	89.7 (88.0;91.1)	12,953	85,4 (83,8;87,0)
≤ 16	10.921	86.1 (83.6;88.3)	9.126	69,6 (66,6;72,4)
Don’t know	836	74.5 (66.2;81.3)	785	72,0 (64,0;78,9)
**Monthly household income**				
Up to BRL 1,000.00	7.619	88.8 (86.7;90.5)	7.233	84,7 (82,7;86,5)
From BRL 1,001.00 to R$ 3,000.00	11.220	89.2 (87.5;90.7)	10,733	86,2 (84,2;88,0)
From BRL 3,001.00 to BRL 8,000.00	6.617	89.6 (87.4;91.4)	6.093	80,9 (77,6;83,8)
From BRL 8,001.00 or more	3,984	83.8 (78.7;87.9)	2,939	61,2 (56,1;66,1)
Unknown	3,624	81.2 (76.5;85.1)	3.229	65,0 (60,6;69,2)
**Use of private service**				
Yes	431	85.7 (78.9;90.5)	5,512	61,4 (57,4;65,2)
No	32,489	87.6 (86.3;88.7)	24.121	84,8 (83,5;86,0)

It is noteworthy that the vaccination coverage of the group in where mothers work (90.2%; 95%CI 88.7;91.4) is higher than that of mothers who do not work (86.0%; 95%CI 84.0;87.7), a pattern also observed for the complete vaccination schedule with the first booster dose, in which there are, respectively, 84.5% (95%CI 82.8;86.1) and 76.0% (95%CI 73.9;78.0) (Table 4).

Regarding the analysis focused on the complete schedule with the first booster dose, it is found that White race/skin color has lower coverage (75.1%; 95%CI 72.6;77.5) compared to Black (87.9%; 95%CI 85.3;90.1) and mixed-race (83.1%; 95%CI 81.4;84.7). In addition, vaccination coverage for consumer goods strata C (85.7%; 95%CI 83.6;87.5) and D (85.0%; 95%CI 83.3;86.7) is higher than that for stratum B (69.8%; 95%CI 66.3;73.0), which in turn is higher than that for stratum A (43.3%; 95%CI 36.2;50.8); as observed with the complete vaccination schedule, it is also found that vaccination coverage for those receiving the Bolsa Família Program benefit (86.8%; 95%CI 85.2;88.2) is higher than for those who do not receive it (77.1%; 95%CI 75.3;78.8) (Table 4).

For the first booster, vaccination coverage is lower for mothers aged 35 or over (74.9%; 95%CI 72.2;77.5). In addition, it was observed that, for mothers without a partner, vaccination coverage is more satisfactory (84.0%; 95%CI 81.7;86.0), compared to those with a partner (78.6%; 95%CI 76.8;80.2). Nevertheless, mothers with 16 years of education or more vaccinate their children less, with coverage of 69.6% (95%CI 66.6;72.4) for this group (Table 4).

It is noteworthy that, for vaccination with a booster dose, the analysis of monthly household income shows that coverage is higher in the group of families with income between BRL1,001.00 to BRL3,000.00 (86.2%; 95%CI 84.2;88.0), which in turn is higher than that of the groups from BRL3,001.00 to BRL8,000.00 (80.9%; 95%CI 77.6;83.8) and BRL8,001.00 or more (61.2%; 95%CI 56.1;66.1). There is also higher coverage in the group that does not use private services (84.8%; 95%CI 83.5;86.0), i.e., those who receive vaccination through the Brazilian National Health System (*Sistema Único de Saúde* - SUS) (Table 4).

## DISCUSSION

This study showed that the estimated coverage for IPV and OPV did not meet the recommended target, with no homogeneity observed in the coverage analyzed,^19^ with lower coverage with booster doses, with a significant reduction in vaccination coverage when considering validity and timeliness.

Regarding the observed OPV vaccination coverage, which was higher than that measured in the SI-PNI,^1^ it is worth highlighting that this result may be directly associated with the data collection method, since checking physical records addresses issues related to the recording of doses administered in the information system, potentially leading to differences between local data and consolidated national figures.^20^

Although no significant differences were observed between socioeconomic strata A, C, and D, where wealthier individuals tend to vaccinate less, a fact also observed in other studies,^21^ it is worth noting that there is a greater possibility of international exposure among wealthier individuals, which could increase the likelihood of infection and the importation of cases, as described in a study on the introduction of COVID-19 in Brazil.²² However, in the face of local transmission, it is understood that, even with more satisfactory coverage among poorer populations, given the existing social conditions and determinants, this population may be more affected, as observed in the COVID-19 pandemic.^22,23^

It is worth highlighting that family, maternal and child characteristics, when analyzed in a univariate manner, only allow for inferences, and it could be seen that the results focused on the vaccination schedule with the first booster dose being more satisfactory in the group with lower-income families may be linked to those who are beneficiaries of the Bolsa Família Program, which also use the SUS for vaccination, which is a requirement of the program itself,^24^ and this initiative can be a key factor in achieving vaccination coverage in Brazil. A study conducted in Brazil and its regions in 2018 shows that the Bolsa Família Program contributed to increasing overall vaccination status among children.^25^

However, considering the immunity conferred by booster doses,^26^ it is crucial to emphasize the importance of a complete vaccination schedule with all recommended doses. Taking into account additional risk factors, such as population density, intermunicipal and interstate flow, migration flows, and the presence of important gateways (ports, airports and bus stations),^27^ underscore the need to increase vaccination coverage in large urban centers.

Furthermore, some states, such as Acre, Amazonas, Goiás, Mato Grosso, Mato Grosso do Sul, Rio Grande do Sul and Paraná, have native populations living in hard-to-reach areas and border populations, making them more vulnerable^5^ and requiring specific strategies to reach these populations.

In this context, the study showed the importance of checking physical vaccination booklets, since they are the primary tool for vaccination verification, in addition to facilitating the understanding of vaccination coverage, they also assist in the process of defining effective strategies to minimize barriers to vaccination.^28^ These strategies must consider existing regional differences and the needs of the most vulnerable populations, and that, in addition to adherence to vaccination, it can be promoted in a timely manner, in order to reduce the number of susceptible people in a given period.

Efforts to minimize vaccine hesitancy are crucial for reducing dropout rates in both complete vaccination schedules and booster doses, given that the WHO has included it in the list of top ten threats to global health, given its potential to reverse progress in combating vaccine-preventable diseases. ^28,29^ Thus, vaccine hesitancy poses a challenge to improving vaccination coverage, one that extends beyond access issues, since this study shows that, for polio coverage, vaccination campaigns were not correlated with high coverage, highlighting the need to assess communication and public engagement strategies, aimed to obtain satisfactory results, similar to those historically attained by the PNI.^30^

The limitations of this survey are related to access to families – due to mistrust, urban insecurity or lack of interest in participating in surveys –, as well as difficulty in reading vaccination booklets and the fact that the census was not conducted in 2020, which led to the use of outdated data to define socioeconomic strata, which may have generated selection bias.^2^

In conclusion, understanding the vaccination status guides decision-making processes in the development of actions to increase vaccination coverage and redefine strategies, such as campaigns. This, in turn, is expected to reduce the number of susceptible individuals, helping control vaccine-preventable diseases and reducing the risk of reintroducing diseases that have been eliminated in certain regions.
