# Supplementary material for: Analysis of polio vaccination status in a cohort of live births in 2017 and 2018 in Brazilian cities: a national vaccination coverage survey
Source: Epidemiol Serv Saude. 2025 Jan 10;33(spe2):e20231303. doi: 10.1590/S2237-96222024v33e20231303.en (PMC11734592; doi:10.1590/S2237-96222024v33e20231303.en)
Supplement: Translation

# Translation: Análise da situação vacinal de poliomielite em coorte de nascidos vivos de 2017 e 2018 em cidades brasileiras: inquérito nacional de cobertura vacinal

**DOI:** 10.1590/S2237-96222024v33e20231303.pt

**Authors:** Alessandra Lucchesi de Menezes Xavier Franco, Ana Paula França, José Cássio de Moraes, Manoel Carlos Sampaio de Almeida Ribeiro, Grupo ICV 2020, Adriana Ilha da Silva, Alberto Novaes Ramos, Ana Paula França, Andrea de Nazaré Marvão Oliveira, Antonio Fernando Boing, Carla Magda Allan Santos Domingues, Consuelo Silva de Oliveira, Ethel Leonor Noia Maciel, Ione Aquemi Guibu, Isabelle Ribeiro Barbosa Mirabal, Jaqueline Caracas Barbosa, Jaqueline Costa Lima, José Cássio de Moraes, Karin Regina Luhm, Karlla Antonieta Amorim Caetano, Luisa Helena de Oliveira Lima, Maria Bernadete de Cerqueira Antunes, Maria da Gloria Teixeira, Maria Denise de Castro Teixeira, Maria Fernanda de Sousa Oliveira Borges, Rejane Christine de Sousa Queiroz, Ricardo Queiroz Gurgel, Rita Barradas Barata, Roberta Nogueira Calandrini de Azevedo, Sandra Maria do Valle Leone de Oliveira, Sheila Araújo Teles, Silvana Granado Nogueira da Gama, Sotero Serrate Mengue, Taynãna César Simões, Valdir Nascimento, Wildo Navegantes de Araújo

**Affiliations:** 1 Centro de Vigilância Epidemiológica “Prof. Alexandre Vranjac”, Coordenadoria de Controle de Doenças, São Paulo, SP, Brasil; 2 Faculdade de Ciências Médicas da Santa Casa de São Paulo, Departamento de Saúde Coletiva, São Paulo, SP, Brasil; 3 Coordenadoria de Controle de Doenças, Secretaria de Estado da Saúde de São Paulo, São Paulo, SP, Brasil; Universidade Federal do Espírito Santo, Vitória, ES, Brasil; Universidade Federal do Ceará, Departamento de Saúde Comunitária, Fortaleza, CE, Brasil; Faculdade Ciências Médicas Santa Casa de São Paulo, São Paulo, SP, Brasil; Secretaria de Estado da Saúde do Amapá, Macapá, AP, Brasil; Universidade Federal de Santa Catarina, SC, Brasil; Organização Pan-Americana da Saúde, Brasília, DF, Brasil; Instituto Evandro Chagas, Belém, PA, Brasil; Faculdade de Ciências Médicas Santa Casa de São Paulo, Departamento de Saúde Coletiva, São Paulo, SP, Brasil; Universidade Federal de Mato Grosso, Cuiabá, MT, Brasil; Universidade Federal do Paraná, Curitiba, PR, Brasil; Universidade Federal de Goiás, Goiânia, GO, Brasil; Universidade Federal do Piauí, Teresina, PI, Brasil; Universidade de Pernambuco, Faculdade de Ciências Médicas, Recife, PE, Brasil; Instituto de Saúde Coletiva, Universidade Federal da Bahia, Salvador, BA, Brasil; Secretaria de Estado da Saúde de Alagoas, Maceió, AL, Brasil; Universidade Federal do Acre, Rio Branco, AC, Brasil; Universidade Federal do Maranhão, Departamento de Saúde Pública, São Luís, MA, Brasil; Universidade Federal de Sergipe, Aracaju, SE, Brasil; Secretaria Municipal de Saúde, Boa Vista, RR, Brasil; Fundação Oswaldo Cruz, Mato Grosso do Sul, Campo Grande, MS, Brasil; Fundação Oswaldo Cruz, Escola Nacional de Saúde Pública Sergio Arouca, Rio de Janeiro, RJ, Brasil; Universidade Federal do Rio Grande do Sul, Porto Alegre, RS, Brasil; Fundação Oswaldo Cruz, Instituto de Pesquisa René Rachou, Belo Horizonte, MG, Brasil; Secretaria de Desenvolvimento Ambiental de Rondônia, Porto Velho, RO, Brasil; Universidade de Brasília, Brasília, DF, Brasil

**Keywords:** Cobertura Vacinal, Vacinas contra Poliovírus, Poliomielite, Saúde da Criança, Inquéritos Epidemiológicos

## Abstract

**Objetivo:**

Descrever a situação vacinal contra poliomielite em 26 capitais, Distrito Federal e 12 municípios do Brasil, em crianças nascidas em 2017 e 2018.

**Métodos:**

Inquérito domiciliar de base populacional realizado de 2020 a 2022, em que se avaliou a cobertura vacinal da poliomielite em crianças, considerando doses válidas, aplicadas e oportunas por municípios.

**Resultados:**

Foram coletados dados de 37.801 crianças. A cobertura vacinal de esquema completo de doses válidas foi de 87,5% (IC_95%_ 86,2;88,7), caindo para 79,6% (IC_95%_ 78,1;81,0), considerando a dose de reforço. A taxa de abandono foi de 4,5% para esquema completo, e de 11,7% no primeiro reforço. Não houve correlação entre a realização de campanha e as elevadas coberturas.

**Conclusão:**

A cobertura vacinal de esquema completo e no primeiro reforço não atingiu a meta de 95,0%. Diferenças regionais e a associação das coberturas vacinais com indicadores sociais devem ser consideradas nas estratégias de aumento da cobertura.

## Contribuições do estudo

**Principais resultados:**

As coberturas vacinais de esquema completo contra poliomielite são de 87,5% (IC_95%_ 86,2;88,7) e observada redução para 79,6% (IC_95%_ 78,1;81,0) com a primeira dose de reforço. Taxa de abandono de 4,5% para esquema completo, e 11,7% no primeiro reforço.

**Implicações para os serviços:**

A verificação das cadernetas de vacinação favorece a identificação e minimização dos fatores associados à não vacinação, que devem ser considerados para a definição de estratégias assertivas que visem ao aumento das coberturas vacinais.

**Perspectivas:**

A compreensão das coberturas vacinais direciona o processo de desenvolvimento de ações que favoreçam o incremento das coberturas vacinais e consequentemente reduzam o número de suscetíveis, auxiliando no controle das doenças imunopreveníveis.

## INTRODUÇÃO

Desde 2016, as coberturas das vacinas do calendário infantil têm apresentado queda, refletindo no resultado insatisfatório desse indicador para a vacina da poliomielite no Brasil. Nos anos de 2020, 2021 e 2022 registrou-se uma cobertura vacinal no país para esquema completo com primeiro reforço de 73,0%, 65,8% e 72,5%, respectivamente, não sendo alcançada a meta preconizada de 95,0%.^1^ Esses resultados podem estar associados a diferentes fatores, tais como operacionais, de agravamento das condições socioeconômicas e de hesitação vacinal.^2^ A introdução da vacina contra a poliomielite no Brasil data de 1961.^3^ No ano de 2016 foi incorporada ao esquema vacinal o uso da Vacina Inativada contra Poliomielite (VIP) com três doses no primeiro ano de vida da criança (aos 2, 4 e 6 meses de idade), e realização de duas doses de reforço com a Vacina Oral de Poliovírus (VOP) bivalente^4^ aos 15 meses e 4 anos de vida, como também sua administração em campanhas anuais de vacinação.^5^ No entanto, o referido esquema vacinal sofrerá mudanças em 2024, já que a Organização Mundial da Saúde (OMS) recomenda utilizar VIP sempre que possível,^6^ e dessa forma as doses de reforço serão realizadas com VIP e não com VOP.^5,7^

Destaca-se que a poliomielite é uma doença infectocontagiosa viral aguda que acomete principalmente crianças menores de 5 anos de idade, e 1% dos casos apresentam paralisia flácida de início súbito em membros inferiores, assimétrica, com sensibilidade preservada, e arreflexia no segmento atingido.^8^ A transmissão ocorre mais frequentemente através do contato direto com fezes ou secreções eliminadas pela boca de pessoas doentes ou portadores.^8,9^ O déficit motor tem evolução frequentemente em três dias, 5% a 10% dos casos morrem por paralisia dos músculos respiratórios^10^ e uma em cada 200 infecções leva à paralisia irreversível.^8^

Ressalta-se que, em 1988, a 41ª Assembleia Mundial da Saúde adotou resolução sobre a erradicação mundial da poliomielite, que marcou a criação da Iniciativa Global de Erradicação da Pólio (GPEI).^11^ Tal iniciativa propiciou a ampliação da capacidade de enfrentamento a outras doenças infectocontagiosas e a qualificação da vigilância e imunização,^12^ o que corroborou para a redução dos casos, tendo em vista que atualmente 80% da população mundial vive em regiões certificadas como livres da pólio.^13^

O Brasil foi certificado pela eliminação da poliomielite em 1994, contudo existe o risco de casos importados,^14^ uma vez que há países com transmissão endêmica, como o Afeganistão e o Paquistão.^15,16^ Não obstante, há mais de 25 países com detecção de poliovírus selvagem do tipo 1 ou derivados da vacina, com risco potencial de propagação internacional.^16^ Ressalta-se que enquanto houver uma criança infectada, há risco de crianças suscetíveis de todos os países contraírem poliomielite^15^ e, portanto, a necessidade de manutenção de coberturas vacinais elevadas,^9,11^ fato que apoia a necessidade da realização de estudos que auxiliem a compreensão da situação vacinal e o direcionamento de ações para melhores resultados. 

O objetivo deste estudo é descrever a situação vacinal da poliomielite em 26 capitais, no Distrito Federal e em 12 municípios do Brasil, em crianças nascidas em 2017 e 2018. 

## MÉTODOS

Trata-se de um inquérito domiciliar de base populacional, a partir de uma coorte de crianças nascidas vivas em 2017 e 2018, e residentes na zona urbana das capitais de 26 estados, no Distrito Federal e em 12 municípios acima de 100 mil habitantes, sendo: Campinas/SP, Caruaru/PE, Imperatriz/MA, Joinville/SC, Londrina/PR, Petrópolis/RJ, Rio Grande/RS, Rio Verde/GO, Rondonópolis/MT, Sete Lagoas/MG, Sobral/BA e Vitória da Conquista/BA.^2^

### Coleta de dados

A coleta de dados foi realizada entre setembro de 2020 e março de 2022, considerando os períodos de distanciamento social aplicados em cada local. Os dados das datas de aplicação de cada vacina ao longo dos primeiros 24 meses de vida foram obtidos considerando as vacinas administradas na rotina, em serviços públicos e privados, e as administradas em campanhas.

As cadernetas de vacinação foram fotografadas, lidas e transcritas para o banco da pesquisa, por enfermeiros com experiência nas atividades do Programa Nacional de Imunizações (PNI). Aquelas não encontradas no domicílio foram buscadas no Sistema de Informação do PNI (SI-PNI). Ainda foi aplicado um questionário estruturado com perguntas fechadas relacionadas a dados sociodemográficos da criança; reprodutivos e sociodemográficos da mãe; domicílio e consumo da família; e relacionados à vacinação da criança. Mais detalhes sobre o instrumento de coleta e estratégias de campo estão descritos no artigo de referência metodológica.^2^

### Amostragem 

A definição da amostra considerou a realização de inquérito para cada cidade. Dessa forma, para o cálculo foram considerados: população hipotética de 1 milhão de nascidos vivos; prevalência estimada de cobertura vacinal = 70%, erro de estimativa = 5%; z = 1,96 para intervalo de confiança de 95% (IC_95%_); e efeito de desenho de 1,4, com os quais se obtém 452 crianças por inquérito.

Conforme o tamanho da população de nascidos vivos, foram realizados de um a quatro inquéritos por município, sendo que em 15 cidades (4 capitais e 11 do interior) foi realizado apenas um inquérito, dois inquéritos em nove capitais, três inquéritos em quatro capitais e quatro inquéritos em nove capitais, no Distrito Federal e em uma cidade do interior.

O procedimento amostral parte da definição dos estratos socioeconômicos criados a partir da análise de *cluster,* com base em indicadores socioeconômicos (renda média dos responsáveis pelo domicílio, proporção de responsáveis alfabetizados e proporção de responsáveis com renda maior ou igual a 20 salários mínimos) dos setores censitários urbanos de cada cidade, segundo informações do censo demográfico de 2010. 

A análise de conglomerados criou quatro estratos (A a D) de setores censitários com distintas características socioeconômicas. O estrato A refere-se ao grupo socioeconômico de renda alta, o B de renda média alta, o C de renda média baixa, e o D de renda baixa.² Foram estimadas as crianças das coortes de interesse residentes em cada setor, por meio de georreferenciamento dos endereços contidos no Sistema de Informação sobre Nascidos Vivos (Sinasc) e da projeção baseada na distribuição observada no Censo de 2010.

Os setores foram agrupados por proximidade (conglomerados) e número de crianças previsto, de modo que cada conglomerado contivesse três vezes o número de crianças a serem incluídas na amostra. Os conglomerados foram sorteados de modo sistemático para percorrer toda a área geográfica. Com os mapas dos conglomerados e a listagem dos endereços, obtidos no Sinasc, os entrevistadores percorreram a área buscando as crianças das coortes até atingir o número preestabelecido, para cada estrato, em cada cidade, conforme descrito no artigo metodológico do estudo.^2^

### Indicadores e análise dos dados

Para o cálculo das coberturas vacinais, foi utilizado como denominador o número de nascidos vivos registrados no Sinasc em 2017 e 2018, para numeradores utilizaram-se critérios diferentes para considerar doses aplicadas (dose administrada independentemente do momento da aplicação), válidas (dose administrada considerando o momento da aplicação, com relação à data de nascimento e observando-se o intervalo entre as doses) e oportunas (doses recomendadas segundo o esquema vacinal, levando-se em conta as idades mínima e máxima de cada vacina e intervalos adequados entre as doses),^17^ detalhadas a seguir:

- Cobertura vacinal aos 12 meses
  - Doses aplicadas
    - Dose 1: junção da primeira dose de VIP + vacina hexavalente (tríplice bacteriana acelular, VIP, hepatite B e *Haemophilus influenzae* b) + acelular (tríplice bacteriana acelular e VIP);
    - Dose 2: junção da segunda dose de VIP + vacina hexavalente + acelular;
    - Dose 3: junção da terceira dose de VIP + vacina hexavalente + acelular.
  - Doses válidas
    - Dose 1: primeira dose aplicada com 42 dias ou mais;
    - Dose 2: segunda dose aplicada com pelo menos 30 dias após primeira dose;
    - Dose 3: terceira dose aplicada com pelo menos 30 dias após segunda dose.
  - Doses oportunas
    - Dose 1: primeira dose aplicada entre 42 e 89 dias;
    - Dose 2: segunda dose aplicada entre 70 e 151 dias;
    - Dose 3: terceira dose aplicada entre 98 e 212 dias.
- Cobertura vacinal considerando a primeira dose de reforço
  - Doses aplicadas
    - Dose 1: junção da primeira dose de VOP + quarta dose aplicada de VIP ≥ 12 meses.
  - Doses válidas de VOP
    - Dose 1: primeira dose aplicada com 365 dias ou mais, tendo tomado as 3 doses VIP anteriores.
  - Doses oportuna de VOP
    - Dose 1: primeira dose aplicada entre 365 e 486 dias, tendo tomado as 3 doses VIP anteriores.

Considerou-se ainda:

- Indicador de taxa de abandono para esquema vacinal VIP completo: diferença entre doses 1 VIP aplicadas e doses 3 VIP aplicadas/doses 1 VIP aplicadas*100.

- Indicador de taxa de abandono para dose de reforço: diferença entre doses 1 VIP aplicadas e doses 1 VOP (primeiro reforço)/doses 1 VIP aplicadas*100.

Os indicadores de cobertura vacinal foram analisados de forma geral considerando-se os diferentes critérios de doses. Para a análise do comportamento da cobertura entre as capitais e os municípios, foram utilizados os indicadores de doses válidas, sendo que esses também foram utilizados na investigação preliminar da associação das coberturas para as vacinas contra poliomielite com fatores socioeconômicos e demográficos.

Para fins de análise de cobertura vacinal, também foram estabelecidos cinco grupos: satisfatório (igual ou superior a 95%); razoável (90% a 94,9%); insatisfatório (80% a 89,9%); crítico (70% a 79,9%); e muito crítico (igual ou inferior a 69,9%). Os referidos grupos foram estabelecidos entendendo-se que coberturas satisfatórias são correspondentes à meta preconizada de 95%,^18^ e os demais grupos abaixo dessa, de modo que à medida em que os valores se distanciam do preconizado, maiores serão os esforços do município para que se atinja a meta, e maior é a população suscetível, consequentemente o risco para adoecimento e transmissão. 

Como a amostra realizada foi estratificada e conglomerada por setor censitário com alocação desproporcional, foi necessário calcular e utilizar pesos amostrais para cada domicílio entrevistado para cálculo das estimativas. As análises foram realizadas aplicando as definições de pesos, estratos e conglomerados para os cálculos das estimativas de cobertura vacinal e respectivos intervalos de confiança de 95% (IC_95%_), assim como correlações entre as coberturas, esquema com primeiro reforço e campanha foram analisadas pelo teste de correlação de Spearman, utilizando-se o SPSS versão 22.

### Aspectos éticos

A pesquisa foi aprovada pelos Comitês de Ética em Pesquisa com Seres Humanos do Instituto de Saúde Coletiva da Universidade Federal da Bahia, sob parecer nº 3.366.818, em 4 de junho de 2019, com Certificado de Apresentação de Apreciação Ética (CAAE) 4306919.5.0000.5030; e da Irmandade da Santa Casa de São Paulo, sob parecer nº 4.380.019, em 4 de novembro de 2020, com CAAE 39412020.0.0000.5479. 

## RESULTADOS

A amostra final consistiu em 37.801 nascidos vivos, após perda correspondente a 6% da amostra total em cada município. Assim, foi observado que as coberturas vacinais com base nas doses aplicadas para VIP (esquema completo), de 88,0% (IC_95%_ 86,7;89,1), e para VOP, de 81,2% (IC_95%_ 79,8;86,7) (primeira dose de reforço), se encontram abaixo dos 95,0% esperados pelo Ministério da Saúde (Tabela 1). 

**Tabela 1:** Indicadores em (%), com intervalos de confiança de 95% (IC 95%), de cobertura vacinal contra poliomielite para esquema completo e com primeiro reforço na coorte de nascidos vivos de 2017 e 2018, nas capitais, cidades e Distrito Federal, 2020-2021 (n= 37.801)

Indicador	Frequência	% (IC_95_ %)
**Doses aplicadas**		
Dose 1 da vacina inativada poliomielite	34.826	92,1 (91,1;93,0)
Dose 3 da vacina inativada poliomielite	33.273	88,0 (86,7;89,1)
Dose 1 da vacina oral poliomielite	30.746	81,2 (79,8;86,7)
**Doses válidas**		
Dose 1 da vacina inativada poliomielite	34.688	91,5 (90,5;92,5)
Dose 3 da vacina inativada poliomielite	33.064	87,5 (86,2;88,7)
Dose 1 da vacina oral poliomielite	30.227	79,6 (78,1;81,0)
**Doses oportunas**		
Doses 1 da vacina inativada poliomielite	31.000	82,7 (81,4;84,0)
Dose 3 da vacina inativada poliomielite	20.333	58,3 (56,5;60,1)
Dose 1 da vacina oral poliomielite	15.045	41,6 (39,9;43,2)

Esse resultado aponta diferença de 6,8 pontos percentuais entre completitude do esquema vacinal e primeiro reforço, preconizado aos 15 meses de vida da criança. Destaca-se que a taxa de abandono para esquema completo VIP de doses aplicadas é 4,5%, inferior à taxa de abandono relacionada ao esquema vacinal com a primeira dose de reforço (11,7%) (Tabela 1).

Ao serem estabelecidos critérios para considerar as doses válidas, há redução nas coberturas para esquema completo da VIP (87,5%; IC_95%_ 86,2;88,7) e VOP (79,6%; IC_95%_ 78,1;81,0). Esse resultado indica maior redução das coberturas com doses válidas e oportunas para as doses VOP em relação às coberturas dessas doses para esquema com doses VIP. Para 1,7% das crianças que receberam VOP, as doses aplicadas não foram consideradas válidas, enquanto para 0,6% das terceiras doses VIP aplicadas não foram consideradas válidas (Tabela 1). 

Ao ser observada a cobertura vacinal com base na oportunidade, há maior redução do que quando observada a cobertura de doses aplicadas e válidas tanto para VIP (58,3%; IC_95%_ 56,5;60,1) quanto para VOP (41,6%; IC_95%_ 39,9;43,2). Afirma-se que, entre as doses aplicadas, 38,9% das terceiras doses de VIP não foram administradas entre 98 e 212 dias após a segunda dose de VIP, e que 51,1% das primeiras doses de reforço com VOP foram administradas entre 365 e 486 dias após a completitude do esquema com VIP (Tabela 1).

Para a cobertura vacinal do primeiro reforço, a meta não foi alcançada em nenhuma das cidades analisadas, sendo a maior cobertura 93,3% (IC_95%_ 90,7;95,2), em Joinville/SC, e a menor 61% (IC_95%_ 57,3;67,9), em Florianópolis/SC (Tabela 2). Nessa perspectiva, considerando os grupos estabelecidos, se observou a concentração de 10,3% dos municípios no grupo razoável, com coberturas vacinais que variaram de 90,5% a 93,3%. No grupo insatisfatório, concentraram-se 53,8% dos municípios, e as coberturas variaram de 80% a 89,1%; no grupo crítico, essas variaram de 70,9% a 79,8%, contando com 30,8% dos municípios nesse grupo. Por fim, no grupo muito crítico, observou-se 5,1% dos munícipios, com coberturas que variaram de 61,0% (IC_95%_ 53,7;67,9) a 62,2% (IC_95%_ 54,3;69,5) (Tabela 2).

**Tabela 2:** Indicador em (%), com intervalos de confiança de 95% (IC_95_), de cobertura vacinal para vacina inativada poliomielite (VIP) e vacina oral poliomielite (VOP) administradas na rotina e em campanha na coorte de nascidos vivos de 2017 e 2018, de acordo com doses válidas por capital, cidades do interior e Distrito Federal, 2020-2021 (n = 37.801)

Cidade	FU	Região	n	Cobertura (dose 3 VIP+ dose 1 VOP)		Campaign	
				Frequência	% (IC_95_)	Frequência	% (IC_95_)
Joinville	SC	S	460	409	93,3 (90,7;95,2)	258	62,2 (53,9;69,8)
Caruaru	PE	NE	462	422	91,8 (86,6;95,1)	302	61,2 (52,8;68-9)
Porto Velho	RO	N	451	398	91,2 (87,5;93,9)	161	39,6 (30,7;49,3)
Teresina	PI	NE	899	771	90,5 (86,1;93,6)	540	60,9 (53,8;67,6)
Imperatriz	MA	NE	465	423	89,1 (84,2;92,7)	190	36,6 (31,6;41,9)
Sete Lagoas	MG	SE	451	411	89,0 (82,8;93,2)	290	63,2 (52,7;72,5)
Londrina	PR	S	455	370	88,9 (81,7;93,5)	227	47,1 (41,5;52,8)
Salvador	BA	NE	1.818	1.550	88,4 (83,9;91,8)	721	43,0 (38,7;47,4)
Boa Vista	RR	N	395	334	88,1 (81,8;92,4)	172	46,9 (41,9;52,0)
Rio Verde	GO	CO	444	391	87,4 (77,2;93,4)	229	51,7 (42,2;61,1)
Cuiabá	MT	CO	814	703	87,3 (81,4;91,6)	411	52,2 (48,0;56,4)
São Luís	MA	NE	854	708	86,8 (81,2;90,9)	362	39,2 (28,8;50,7)
Sobral	CE	NE	465	356	85,9 (71,5;93,7)	225	67,2 (47,3;82,3)
Manaus	AM	N	1.826	1.546	85,8 (82,1;88,9)	988	55,7 (50,5;60,8)
Porto Alegre	RS	S	1.383	1.012	84,2 (78,8;88,4)	465	41,1 (35,4;47,0)
Rio Grande	RS	S	452	352	84,0 (72,2;91,4)	203	46,7 (31,0;63,1)
Palmas	TO	N	453	354	83,8 (77,3;88,7)	243	54,4 (47,4;61,2)
Petrópolis	RJ	SE	468	415	82,8 (70,5;90,7)	236	44,4 (30,4;59,3)
Aracaju	SE	NE	900	733	82,7 (75,3;88,2)	552	62,4 (56,6;67,9)
Rio Branco	AC	N	451	361	82,7 (78,2;86,5)	160	34,1 (25,9;43,5)
Vitoria da Conquista	BA	NE	455	336	82,1 (67,1;91,2)	193	50,0 (37,4;62,6)
Rondonópolis	MT	CO	449	353	81,3 (72,2;87,9)	196	46,2 (39,2;53,5)
Goiânia	GO	CO	1.811	1.453	81,2 (73,8;86,9)	871	50,1 (42,8;573)
Brasília	DF	CO	1.809	1.362	80,9 (77,3;84,1)	980	57,8 (53,5;62,1)
Maceió	AL	NE	929	744	80,0 (67,2;88,7)	466	46,7 (39,1;54,4)
Fortaleza	CE	NE	1.612	1.328	79,8 (74,3;84,3)	830	51,9 (47,0;56,7)
Belo Horizonte	MG	SE	1.863	1.404	78,9 (74,0;83,1)	1.011	59,5 (54,6;64,2)
Recife	PE	NE	1.689	1.381	78,9 (69,0;86,2)	940	53,6 (45,2;61,7)
São Paulo	SP	SE	1.539	1.250	77,8 (72,8;82,1)	669	44,0 (38,7;49,4)
Campo Grande	MS	CO	1.281	994	77,3 (71,2;82,4)	593	48,6 (42,7;54,5)
João Pessoa	PB	NE	904	705	75,8 (68,8;81,6)	492	53,0 (47,4;58,4)
Rio de Janeiro	RJ	SE	1.820	1.351	74,9 (70,3;78,9)	767	44,8 (39,0;50,8)
Campinas	SP	SE	1.774	1.481	74,5 (62,4;83,8)	826	42,8 (35,0;51,0)
Belém	PA	N	1.218	1.008	73,7 (63,7;81,7)	539	35,5 (27,7;44,1)
Natal	RN	NE	685	530	71,8 (60,5;80,9)	348	47,7 (39,7;55,9)
Curitiba	PR	S	1.192	880	71,5 (62,7;78,9)	538	50,9 (43,6;58,1)
Macapá	AP	N	878	624	70,9 (66,4;75,0)	238	26,2 (20,7;32,6)
Vitória	ES	SE	788	504	62,2 (54,3;69,5)	458	44,6 (29,9;60,2)
Florianópolis	SC	S	739	520	61,0 (53,7;67,9)	291	32,6 (26,3;39,6)

As coberturas vacinais para dose de reforço, nos 12 grandes centros urbanos (São Paulo/SP, Brasília/DF, Rio de Janeiro/RJ, Manaus/AM, Belém/PA, Fortaleza/CE, Recife/PE, Salvador/BA, Belo Horizonte/MG, Curitiba/PR, Goiânia/GO e Porto Alegre/RS), variou de 71,5% (IC_95%_ 62,7;78,9) a 88,4% (IC_95%_ 83,9;91,8), com maior concentração desses centros no grupo crítico (Tabela 2).

Não foi possível observar elevadas coberturas de doses de campanha (Tabela 2), e o dados mostram que, após a realização do teste estatístico, não houve correlação entre a realização de campanha e as elevadas coberturas vacinais.

Quando observados os estratos socioeconômicos, nenhum atingiu a cobertura vacinal preconizada, sendo o estrato C com a melhor cobertura para VIP válidas (89,1%; IC_95%_ 87,4;90,7), contudo, o estrato D apresentou melhor cobertura para esquema vacinal com o primeiro reforço (84,8%; IC_95%_ 83,0;86,4), logo, superiores ao estrato A [esquema completo – 79,1% (IC_95%_ 74,2;83,2) e com primeiro reforço – 64,7% (IC_95%_ 60,0;69,2)]. Ademais, infere-se que a cobertura da dose de reforço variou mais entre os estratos [64,7% (IC_95%_ 60,0;69,2) a 84,8% (IC_95%_ 83,0;86,4)] do que as coberturas de VIP [79,1% (IC_95%_ 74,2;83,2) a 89,1% (IC_95%_ 87,4;90,7)] (Tabela 3). 

**Tabela 3:** Indicadores em (%), com intervalos de confiança de 95% (IC_95_), de cobertura vacinal para vacina inativada poliomielite (VIP) e vacina oral poliomielite (VOP) na coorte de nascidos vivos de 2017 e 2018 no conjunto das capitais, cidades do interior e Distrito Federal, segundo estratos socioeconômicos, 2020-2021 (n = 37.801)

Estrato	n	Dose 3 VIP válidas		Dose 3 VIP válidas + VOP válidas	
		Frequência	Cobertura (IC_95%_)	Frequência	Cobertura (IC_95%_)
A	8.333	7.067	79,1 (74,2;83,2)	6.062	64,7 (60,0;69,2)
B	9.418	8.165	86,5 (82,9;89,4)	7.336	68,8 (63,9;73,3)
C	9.992	8.910	89,1 (87,4;90,7)	8.347	80,9 (78,4;83,1)
D	10.058	8.922	88,9 (87,1;90,5)	8.482	84,8 (83,0;86,4)

Nesse contexto, ao analisarmos as características da família, da mãe e da criança, infere-se que, para o esquema completo de VIP, ao considerarmos os intervalos de confiança, coberturas vacinais do estrato bens de consumo C (89,1%; IC_95%_ 87,3;90,7) e D (88,2%; IC_95%_ 86,4;89,7) são maiores que as do estrato B (87,3%; IC_95%_ 84,6;89,6), que por sua vez é maior que o estrato A (81,8%; IC_95%_ 75,7;86,7). Nota-se que a cobertura vacinal para quem recebe o benefício do Bolsa Família (90,9%; IC_95%_ 89,3;92,3) é melhor do que para os que não o recebem (86,3%; IC_95%_ 84,8;87,7) (Tabela 4). 

**Tabela 4:** Indicadores em (%), com intervalos de confiança de 95% (IC^95%^), de cobertura vacinal para vacina inativada poliomielite (VIP) e vacina oral poliomielite (VOP) na coorte de nascidos vivos de 2017 e 2018 no conjunto das capitais, cidades e Distrito Federal, segundo as características da família, da mãe e da criança, 2020-2021 (n = 37.801)

Características da família, da mãe e da criança	Dose 3 VIP válidas		Dose 1 VOP válidas	
	Frequência	% (IC^95%^)	Frequência	% (IC^95%^)
Raça/cor da pele da mãe				
Branca	13.195	87,0 (84,8;88,9)	11.471	75,1 (72,6;77,5)
Preta	3.763	89,4 (86,7;91,6)	3.611	87,9 (85,3;90,1)
Parda	14.909	88,3 (86,5;89,9)	14.054	83,1 (81,4;84,7)
Amarela	324	89,4 (78,1;95,2)	275	70,0 (53,8;82,4)
Indígena	111	90,0 (78,2;95,8)	101	85,3 (74,2;92,1)
Sem informação	762	72,6 (63,4;80,2)	715	68,6 (59,8;76,3)
**Estrato bens de consumo**				
A	1.622	81,8 (75,7;86,7)	1.066	43,3 (36,2;50,8)
B	7.822	87,3 (84,6;89,6)	6.621	69,8 (66,3;73,0)
C	10.318	89,1 (87,3;90,7)	9.923	85,7 (83,6;87,5)
D	12.321	88,2 (86,4;89,7)	11.714	85,0 (83,3;86,7)
Sem informação	981	72,7 (64,5;79,6)	903	68,1 (59,5;75,6)
**Bolsa Família**				
Sim	9.089	90,9 (89,3;92,3)	8.662	86,8 (85,2;88,2)
Não	23.866	86,3 (84,8;87,7)	21.457	77,1 (75,3;78,8)
Sem informação	109	87,2 (75,7;93,8)	108	86,3 (76,0;92,6)
**Sexo da criança**				
Masculino	16.993	87,5 (86,0;88,9)	15.540	80,5 (78,7;82,1)
Feminino	16.071	87,5 (85,8;89,0)	14.687	78,7 (76,6;80,7)
**Ordem de nascimento**				
Primeiro	16.013	88,2 (86,5;89,6)	14.584	79,3 (77,2;81,3)
Segundo	10.613	86,4 (84,3;88,3)	9.667	80,0 (77,7;82,2)
Terceiro	4.084	88,3 (85,8;90,5)	3.796	79,6 (76,1;82,7)
Quarto ou mais	2.331	87,0 (82,8;90,2)	2.156	79,6 (74,3;84,1)
**Faixa etária da mãe (anos)**				
< 20	765	87,4 (80,4;92,1)	729	85,5 (79,6;89,9)
20-34	18.973	86,9 (85,3;88,3)	17.921	83,0 (81,4;84,4)
≥ 35	13.182	88,4 (86,6;90,0)	11.442	74,9 (72,2;77,5)
**Companheiro**				
Sim	24.820	87,8 (86,4;89,1)	22.450	78,6 (76,8;80,2)
Não	7.327	87,9 (85,8;89,7)	6.909	84,0 (81,7;86,0)
Não informou	917	76,4 (68,3;82,9)	868	73,2 (65,2;79,9)
**Avó mora junto**				
Sim	8.704	86,2 (84,0;88,1)	8.251	81,4 (79,0;83,7)
Não	24.314	88,0 (86,6;89,3)	21.936	78,9 (77,1;80,6)
**Mãe trabalha**				
Sim	13.506	90,2 (88,7;91,4)	13.666	84,5 (82,8;86,1)
Não	16.539	86,0 (84,0;87,7)	15.863	76,0 (73,9;78,0)
Não informou	735	73,5 (64,3;81,0)	698	69,7 (60,9;77,3)
**Escolaridade da mãe (anos)**				
≤ 8	2.871	87,5 (84,5;90,0)	2.736	82,3 (78,6;85,4)
9-12	4.828	87,0 (84,1;89,5)	4.627	84,6 (81,7;87,1)
13-15	13.608	89,7 (88,0;91,1)	12.953	85,4 (83,8;87,0)
≥ 16	10.921	86,1 (83,6;88,3)	9.126	69,6 (66,6;72,4)
Não sabe	836	74,5 (66,2;81,3)	785	72,0 (64,0;78,9)
**Renda familiar mensal**				
Até R$ 1.000,00	7.619	88,8 (86,7;90,5)	7.233	84,7 (82,7;86,5)
De R$ 1.001,00 a R$ 3.000,00	11.220	89,2 (87,5;90,7)	10.733	86,2 (84,2;88,0)
De R$ 3.001,00 a R$ 8.000,00	6.617	89,6 (87,4;91,4)	6.093	80,9 (77,6;83,8)
De R$ 8.001,00 ou mais	3.984	83,8 (78,7;87,9)	2.939	61,2 (56,1;66,1)
Sem informação	3.624	81,2 (76,5;85,1)	3.229	65,0 (60,6;69,2)
**Uso de serviço privado**				
Sim	431	85,7 (78,9;90,5)	5.512	61,4 (57,4;65,2)
Não	32.489	87,6 (86,3;88,7)	24.121	84,8 (83,5;86,0)

Destaca-se que as coberturas vacinais do grupo em que as mães trabalham (90,2%; IC_95%_ 88,7;91,4) é superior ao das mães que não trabalham (86,0%; IC_95%_ 84,0;87,7), comportamento também observado para o esquema vacinal completo com primeira dose de reforço, em que se tem, respectivamente, 84,5% (IC_95%_ 82,8;86,1) e 76,0% (IC_95%_ 73,9;78,0) (Tabela 4).

Ainda quanto à análise voltada ao esquema completo com primeira dose de reforço, afirma-se que há cobertura vacinal inferior para raça/cor da pele branca (75,1%; IC_95%_ 72,6;77,5) em relação à preta (87,9%; IC_95%_ 85,3;90,1) e à parda (83,1%; IC_95%_ 81,4;84,7). Além disso, a cobertura vacinal do estrato bens de consumo C (85,7%; IC_95%_ 83,6;87,5) e D (85,0%; IC_95%_ 83,3;86,7) é maior que a do estrato B (69,8%; IC_95%_ 66,3;73,0), que por sua vez é maior que a do estrato A (43,3%; IC_95%_ 36,2;50,8); como verificado para o esquema vacinal completo, também se observa que a cobertura vacinal para quem recebe o benefício do Bolsa Família (86,8%; IC_95%_ 85,2;88,2) é melhor do que para os que não o recebem (77,1%; IC_95%_ 75,3;78,8) (Tabela 4).

Para o primeiro reforço, as coberturas vacinais são menores para as mães com 35 anos ou mais (74,9%; IC_95%_ 72,2;77,5). Também se observa que, para mães que não possuem um companheiro, a cobertura vacinal é mais satisfatória (84,0%; IC_95%_ 81,7;86,0), em relação às que possuem um companheiro (78,6%; IC_95%_ 76,8;80,2). Não obstante, mães com 16 ou mais anos de estudo vacinam menos seus filhos, pois foi verificada cobertura de 69,6% (IC_95%_ 66,6;72,4) para esse grupo (Tabela 4).

Ressalta-se que para a vacinação com dose de reforço a renda familiar mensal mostrou que a cobertura é maior no grupo de família com renda de R$ 1.001,00 a R$ 3.000,00 (86,2%; IC_95%_ 84,2;88,0), que por sua vez é maior que a dos grupos de R$ 3.001,00 a R$ 8.000,00 (80,9%; IC_95%_ 77,6;83,8) e R$ 8.001,00 ou mais (61,2%; IC_95%_ 56,1;66,1). Como também se destaca cobertura superior ao grupo que não se utiliza de serviço privado (84,8%; IC_95%_ 83,5;86,0), ou seja, que realiza vacinação através do Sistema Único de Saúde (SUS) (Tabela 4). 

## DISCUSSÃO

O estudo mostrou que a cobertura estimada de VIP e VOP não superou a preconizada, não havendo homogeneidade nas coberturas analisadas,^19^ sendo menor a cobertura com dose de reforço, com redução significativa das coberturas vacinais ao considerar validade e oportunidade.

Sobre a cobertura vacinal com VOP observada, a qual apresenta-se maior do que a aferida no SI-PNI,^1^ destaca-se que tal resultado pode estar diretamente associado à forma de coleta de dados, uma vez que a verificação do registro físico supera problemas relacionados ao registro da dose administrada no sistema de informação, o que pode resultar em diferenças entre os dados locais e os números consolidados em nível nacional.^20^

Muito embora não tenham sido observadas diferenças expressivas entre os estratos socioeconômicos A, C e D, em que se observa que os mais ricos se vacinam menos, fato também observado em outros estudos,^21^ destaca-se que há maior possibilidade de exposição dos mais ricos em território internacional, o que contribui para maior possibilidade de infecção com importação de casos, como descrito em estudo que versa sobre a introdução da covid-19 no Brasil.²² No entanto, diante de transmissão local, entende-se que mesmo com coberturas mais satisfatórias entre os mais pobres, dados os condicionantes e determinantes sociais existentes, há possibilidade de ser a população mais afetada, como observado na pandemia de covid-19.^22,23^

É importante destacar que as características da família, da mãe e da criança, quando analisadas de forma univariada, permitem realizar apenas inferências, e atribui-se que os resultados, voltados ao esquema vacinal com a primeira dose de reforço mais satisfatório no grupo com menor renda familiar, podem estar atrelados às famílias que recebem o benefício do Bolsa Família, que por sua vez fazem uso do SUS para acesso à vacinação, a qual é pré-requisito do próprio programa,^24^ podendo ser indicada como importante iniciativa para o alcance das coberturas vacinais no Brasil. Estudo realizado no Brasil e suas regiões em 2018 aponta que o Programa Bolsa Família aumentou o estado de vacinação das crianças de um modo geral.^25^

Não obstante, considerando a imunidade conferida a partir das doses de reforço,^26^ destaca-se a importância do esquema vacinal completo com todas as doses recomendadas. Considerando ainda fatores que agregam risco adicional, como adensamento populacional, fluxo intermunicipal e interestadual, fluxo migratório, e a presença de pontos de entrada importantes (portos, aeroportos e rodoviárias),^27^ faz-se necessário o incremento das coberturas vacinais nos grandes centros urbanos.

Ademais, alguns estados, como Acre, Amazonas, Goiás, Mato Grosso, Mato Grosso do Sul, Rio Grande do Sul e Paraná, possuem populações originárias vivendo em áreas de difícil acesso e população de fronteira, e são mais vulneráveis,^5^ portanto, demandam estratégias específicas para alcance desse público. 

Nesse sentido, o estudo mostrou a importância da verificação das cadernetas de vacinação em sua forma física, visto que são o principal instrumento para comprovação vacinal, pois além de favorecer a compreensão das coberturas vacinais também auxiliam no processo de definição de estratégias assertivas para minimização dos entraves para vacinação em si.^28^ Estratégias essas que devem considerar as diferenças regionais e as necessidades das populações mais vulneráveis, que, para além da adesão à vacinação, a promovam em tempo oportuno, para redução do número de pessoas suscetíveis em determinado período.

Aspectos relacionados à hesitação vacinal precisam ser minimizados para que as taxas de abandono de esquema vacinal completo e com dose de reforço possam se reduzidas, uma vez que a OMS a incluiu na lista dos dez maiores riscos à saúde global, pela ameaça de reverter o avanço no combate às doenças imunopreveníveis.^28,29^ Nesse sentido, hesitação vacinal constitui um desafio para a melhoria das coberturas vacinais, a ser considerado para além das questões de acesso, já que aponta que para a cobertura de poliomielite e a realização de campanhas não há correlação com elevadas coberturas vacinais, o que indica a necessidade de avaliação dos formatos adotados para comunicação e sensibilização da população, buscando obter resultados satisfatórios como os consagrados ao longo da história do PNI.^30^

As limitações deste inquérito se relacionam ao acesso às famílias, em função da desconfiança, da insegurança urbana ou do desinteresse em participar das pesquisas, às dificuldades de leitura das cadernetas de vacinação e à não realização do censo em 2020, pois foram utilizados dados antigos para a definição dos estratos socioeconômicos, o que pode ter culminado em viés de seleção.^2^

Assim, infere-se que a compreensão da situação vacinal direciona o processo de tomada de decisão no desenvolvimento de ações para o incremento das coberturas vacinais e a redefinição de estratégias utilizadas, a exemplo das campanhas, reduzam o número de suscetíveis, auxiliando no controle das doenças imunopreveníveis, reduzindo o risco de reintrodução de doenças em territórios nos quais foram eliminadas.
